# Analysis of the structure and robustness of the global semiconductor trade network

**DOI:** 10.1371/journal.pone.0313162

**Published:** 2025-01-09

**Authors:** Long Li, Hua Wang, Zhiyi Li, Shaodong Hu

**Affiliations:** 1 Graduate Institute for Taiwan Studies, Xiamen University, Xiamen, Fujian, China; 2 Shantou University Business School, Shantou University, Shantou, Guangdong, China; HJNU: Hanjiang Normal University, CHINA

## Abstract

Amidst the global restructuring of the semiconductor supply chain, this paper constructs a global semiconductor trade network (2007, 2012, 2017, 2021) encompassing three segments (raw materials, equipment, and finished components), based on the CEPII database. After initially exploring trade flows among different regions, the paper conducts an in-depth analysis of the network’s overall structure and the significance of its nodes. Furthermore, the evolution of the trade network’s community structure is discussed and its robustness and dynamics over recent years are assessed through computer program simulation. The findings are as follows: First, semiconductor trade flows are concentrated primarily among a few regions in Asia, US, and EU. Second, the network has grown in size and exhibits significant “small-world” characteristics in all segments, deviating from the typical "sparsity" seen in large-scale networks. Third, Japan, the US, and a few European regions wield significant influence in semiconductor materials and equipment trade, while Asian economies such as Chinese mainland, Chinese Taiwan, and Korea dominate semiconductor components trade. Fourth, the raw materials trade network has diversified in recent years, while the trade networks for equipment and finished components remain in a state of continuous “polarization.” Fifth, the semiconductor trade network demonstrates robustness against random attacks but collapses quickly under targeted attacks. Among the three segments, the trade network of finished components, being larger in scale, exhibits greater resilience against both random and targeted attacks. This paper not only enhances the construction of the global semiconductor trade network but also introduces a dynamic perspective, offering deeper insights into its structure and robustness. The insights gained from this analysis provide valuable guidance for policymakers and companies, especially amidst rapid technological change and geopolitical tensions.

## 1. Introduction

Semiconductors, among the most complex and sophisticated products today, incorporate cutting-edge technologies from various disciplines. They are widely used in critical fields such as computers, consumer electronics, communications, automobiles, and industrial control, serving as the cornerstone of the information technology (IT) industry and the digital economy. The development level of the semiconductor industry chain has become a crucial indicator of a country’s scientific, technological, and industrial strength. In recent years, amidst the global trend of supply chain geopolitics and the push for shorter supply chains, major economies increasingly consider the semiconductor industry vital for economic and security competition [1]. The semiconductor industry has emerged as a focal point in the strategic competition between China and the United States (US). In August 2022, the US took the lead in releasing the “Chip Act” to strategize the semiconductor industry chain. China is also actively developing its semiconductor industry to gain greater control over core technologies and ensure the security of its industrial and supply chains.

The global semiconductor industry originated in the United States in the mid-20th century, underwent significant technological advancements there, and later shifted to Japan in the PC era (1980s). Subsequently, during the Internet era (1990s), it moved to Korea and Chinese Taiwan. The semiconductor industry in Chinese mainland began to emerge after entering the mobile Internet era in the 2010s. Along with the development of industrial technology and the evolution of industrial layout, the division of labor within the global semiconductor industry has been continuously refined. No single company or country can cover the entire industrial chain.

The Integrated Device Manufacturer (IDM) model has been transformed into the Fabless model, and the foundry model, represented by Taiwan Semiconductor Manufacturing Company (TSMC), has risen to prominence. Meanwhile, the global semiconductor market has expanded from approximately $2.9 billion in 1976 to $583.2 billion in 2022, with projections indicating a further increase to $602 billion by 2024. In terms of the upstream and downstream segments of the semiconductor industry chain, the current division of labor within the global semiconductor industry is primarily influenced by the factor endowment advantages of each region. In general, US, the European Union (EU), and Japan are more involved in the upstream links, including design, key materials, and equipment, while the Asia-Pacific region, including Chinese Taiwan, Korea, and Chinese mainland, are more involved in the midstream and downstream links of foundry manufacturing and packaging and testing.

Amid the strategic rivalry between China and the US, the disruptions caused by the COVID-19 pandemic, and the ongoing Russia-Ukraine conflict, the global supply chain is experiencing unprecedented challenges. The substantial scale, intricate complexity, and critical strategic importance of semiconductor trade make it an essential case study for assessing potential vulnerabilities in global supply chains. Against this backdrop, this paper aims to address several questions: What are the current characteristics of global semiconductor trade? What roles do various countries or regions play within this trade network? And how resilient is this trade network to shocks? Answering these questions is essential, as the insights derived from this type of research are crucial for ensuring the security and promoting the sustainable development of the global semiconductor supply chain.

## 2. Literature review

Snyder and Kick pioneered the application of social network analysis to trade research by constructing multiple cross-country interaction networks [2]. Their work delved into each country’s positioning and economic growth within the global trading system, marking the initiation of trade network studies.

Subsequently, Fagiolo et al. significantly advanced trade network research by introducing an innovative construction method for weighted and directional networks [3]. Their approach considered the strength and directionality of connections among nodes, providing a more accurate reflection of interaction strength and influence magnitude within the network. This is crucial for understanding the structure and dynamic characteristics of the network. As scholarly interest grew, researchers increasingly focused on analyzing the global trade network structure, uncovering characteristics such as core–periphery structure [4], high connectivity and density [5], and multiple cores [6].

After the 2008 financial crisis, the focus shifted to the stability of trade networks under uncertainty shocks, introducing the concept of complex network robustness into trade network research. Network robustness is the ability to maintain structural integrity and functionality after random failures or deliberate attacks on nodes or edges. Foti et al. pioneered the introduction of extinction analysis for ecosystems into trading systems, revealing the "robust yet fragile" nature of trade networks [7]. While trade networks demonstrated strong robustness against random attacks, deliberate attacks targeting specific nodes and edges exposed their fragility, diminishing the stability of trade networks [8]. With global political and economic instability escalating, there is renewed interest in researching trade network robustness. Xie et al. made a significant contribution by discovering that simulated intentional attacks, such as state bankruptcies, trade blockades, and economic sanctions, have a more substantial impact on network robustness than random attacks. Notably, the international oil trading system is increasingly vulnerable [9]. Existing studies have predominantly focused on various commodities like food [10], pesticides [11, 12], and minerals [13–16], yet there remains insufficient attention given to high-tech products.

In light of the increasing significance of semiconductors in national economies and international competition, recent studies have conducted a more comprehensive exploration of the structure and robustness of the semiconductor trade. Ren et al. investigated the evolution of cross-border trade flows and dependencies in the global semiconductor trade network [17]. The findings reveal a highly spatially unbalanced semiconductor trade that tends to intensify. Specifically, the trade of manufactured goods and materials has formed a circular system in East Asia, while the trade of semiconductor equipment has established a distinct "production-consumption" boundary on a global scale. Another analysis by Zhang and Zhu examines the structure of the global semiconductor trade network in 2020 and the change in China’s position before and after the US-China trade war [18]. Unlike previous studies [19–21], employing a complex network model to analyze the global semiconductor trade delivers a more systematic approach, providing better insights into the trade’s complex dynamics and evolutionary processes. Furthermore, from an industry chain perspective, this approach integrates the interdependence between products and the complexity of the industry chain, offering a more realistic representation compared to the analysis focused on a single product trade network. However, the following research gaps still exist: (1) Some essential products have not been considered in existing research. Taking optical components as an example, elements such as lasers, optical amplifiers, and optical detectors play pivotal roles in various facets of the semiconductor industry, including manufacturing, measurement, alignment, and communication. (2) Opportunities for refining the global semiconductor trade network topology analysis exist. By introducing a dynamic perspective to scrutinize the trade network’s overall structure and node influence, utilizing chord diagrams for trade flow analysis, a more profound comprehension of global semiconductor trade may be attained, potentially leading to novel discoveries. (3) In conclusion, extant studies into the robustness of semiconductor trade networks reveal potential areas for optimization. These studies not only lack dynamic analysis in the time dimension but also require further discussion on the measurement of network efficiency in computer program simulations. Directly using network efficiency as the metric for network performance would overlook the trade volume information between nodes, which is crucial for providing economic insights.

This paper refines the construction of the semiconductor trade network from an industry chain perspective, focusing on nearly 30 key commodities across three segments: semiconductor raw materials, equipment, and finished components. Utilizing multi-period trade data, this paper provides a comprehensive analysis of the network’s trade flows, overall structure, node significance, community structure, and the robustness of the trade network. Specifically, building upon Ren et al. [17], this paper employs chord diagrams to analyze the fundamental patterns of the trade network and comprehensively explores the characteristics of semiconductor trade by combining network-level metrics and node importance metrics. Secondly, the paper adopts the “cluster Louvain” algorithm for network community detection and supplements the analysis of the evolution of community structure in the semiconductor trade network, providing a new perspective for revealing the development trends of global semiconductor trade. Moreover, based on Zhang and Zhu [18], this paper refines the robustness assessment process by incorporating not only a dynamic perspective but also improving some technical details, such as measuring network performance from the perspective of trade volume for clearer economic implications and repeating multiple attack strategies to validate the results.

This paper is organized as follows: Section 3 details the design of the empirical paper conducted for this analysis. Section 4 provides a baseline description of the current global semiconductor trade network structure. Section 5 analyzes the structural evolution of the trade network, focusing on overall structure, node importance, and community structure. Section 6 evaluates the robustness of the trade network to potential disruptions. Section 7 concludes the paper by summarizing the key findings and discussing the implications.

## 3. Empirical research design

### 3.1. Study subject and data

Within the industrial sector, semiconductors are broadly categorized into two main types: “integrated circuits” (chips) and “optoelectronics, sensors, and discrete semiconductors” (OSD). The semiconductor industry chain comprises design, materials, equipment, manufacturing, packaging and testing. This paper focuses on materials, equipment, and manufacturing due to data availability and their sensitivity to supply chain disruptions (Table 1). The paper relies on the CEPII BACI trade database [22], which provides detailed 6-digit trade data for over 200 economies and more than 5,000 products based on the Harmonized System (HS) code. The dataset spans from 2007 to 2021. This paper avoids aggregation due to potential discrepancies arising from tariffs across Chinese mainland, Hong Kong, Macau, and Chinese Taiwan to ensure the accuracy of trade data analysis for China.

**Table 1:** Semiconductor merchandise trade from an industrial chain perspective.

Process	Commodity	HS code	Describe
Segment 1: Raw materials	Electronic-grade silicon materials	381800 280461 280490	Monocrystalline silicon slices, silicon rods, monocrystalline silicon, compound silicon crystals, selenium crystal rods, etc.
	Rare gases	280429	Rare gases, excluding argon
	Photoresist	370710	Photographic goods; Sensitizing emulsions, put up in measured portions or put up for retail sale in a form ready for use
	Optical elements	900120 900190 900220	Polarizing materials (sheets and plates), unmounted optical elements (lenses, prisms, mirrors) made from various materials (excluding non-optically worked glass), mounted filters used with instruments or equipment (excluding non-optically worked glass)
Segment 2: Equipment	Mounted optical elements for instruments and apparatus	900290	Excluding non-optically worked glass
	Semiconductor inspection equipment	903082 903141	Used for measuring or checking semiconductor wafers or devices, optical instruments and appliances for inspecting semiconductor wafers or devices, or for inspecting photomasks or reticles used in manufacturing semiconductor devices
	Semiconductor wafer fabrication equipment	848610	Used solely or principally for the manufacture of semiconductor boules or wafers
	Semiconductor manufacturing precision machinery	848620	Used solely or principally for the manufacture of semiconductor devices or of electronic integrated circuits
	Flat panel display manufacturing equipment	848630	Used solely or principally for the manufacture of flat panel displays
	Specialized equipment for semiconductor assembly and processing	848640	Used solely or principally for the manufacture or repair of masks and reticles, assembling semiconductor devices or electronic integrated circuits, or for lifting, handling, loading, or unloading items of heading 8486
	Unspecified parts and accessories of machinery or mechanical appliances	848690	Machines and apparatus of heading 8486; parts and accessories
Segment 3: Finished components	Electronic integrated circuits	8542	Integrated circuits and microelectronic component parts such as CPU, GPU, chips, etc.
	Solid-State Non-Volatile Storage Devices	852351 852352 852359	Solid-state non-volatile storage devices(flash memory, PROM, etc.), smart cards, other non-volatile storage
	Capacitor parts, resistor parts, printed circuits	853290 8533 8534	Capacitors, resistors, printed circuit boards, and other semiconductor electrical components of different types and functions
	Vacuum tubes, diodes, transistors	8540 854110 854121 854129	Semiconductor electrical components such as tubes, diodes, and transistors of different types and functions
	Signal & power control with semiconductors	854130 854151	Capacitors, resistors, printed circuit boards, and other semiconductor electrical components of different types and functions

### 3.2. Research methodology

#### 3.2.1. Construction of the global semiconductor trade network

To construct a comprehensive semiconductor trade network, precise identification of the goods exchanged in each trade link is essential. This paper leverages relevant academic research [17, 23, 24], industry reports, and expert consultations to identify specific goods within each segment of the semiconductor trade, linking them to corresponding Harmonized System (HS) codes. Considering significant events such as the 2008 financial crisis, the 2018 US-China trade war, and the 2020 pandemic’s impact on global trade, and adhering to the principle of equidistant sampling, the years designates 2007, 2012, 2017, and 2021 were designated as the time nodes for trade network analysis. Specifically, goods from the aforementioned semiconductor production segments were selected, and the network construction method proposed by Fagiolo et al. was applied to establish a weighted and directed international trade flow matrix *M* [3], where *M* = (*V_i_,V_j_,W,A*), for semiconductor trade between different countries (regions). In this matrix, assuming there are *n* economies, vectors *V*_*i*_ (*i = 1*,*2*,*···*,*n*) and *V*_*j*_ (*j = 1*,*2*,*···*,*n*) represent the exporting and importing locations in the trade network, respectively. The weight matrix *W =* [*W*_*ij*_] (*i = 1*,*2*,*···*,*n; j = 1*,*2*,*···*,*n*) represents the trade value of exports from region *i* to region *j*, while the adjacency matrix *A* = [*A*_ij_] (*i = 1*,*2*,*···*,*n; j = 1*,*2*,*···*,*n*) indicates the presence of an export relationship between region *i* and region *j*.

#### 3.2.2 Network metric analysis

To characterize the overall structure of the global semiconductor trade network, various perspectives are selected for constructing corresponding measurement frameworks, drawing on relevant literature (Table 2). These perspectives include network size, distance [25], convenience [26], and connectivity [27]. These metrics reveal topological structural information about the global semiconductor trade network from diverse angles, offering valuable insights into its evolution. To illustrate the role of nodes within the global semiconductor trade network, measurement frameworks (Table 3) are developed based on factors such as nodes’ direct influence [28], intermediary influence [29, 30], and comprehensive influence [31]. These metrics illuminate the roles of different regions within the trade network. In terms of implementation, the “igraph” package in R was utilized to analyze the overall network structure and node importance, and the “circlize” package was employed to visualize trade flows.

**Table 2:** Metrics system for analyzing the overall structure of the network.

Perspective	Network Metrics	Evaluation
Size	Number of nodes (+); Number of edges (+); Total trade (+)	The size of trade networks
Distance	Network Diameter (-)	The maximum value of the distance between any two nodes in the network, “small-world”
Convenience	Average path length (-)	The average of the shortest distance between any two nodes in the network
Connectivity	Network Density (+)	The density of the ties between nodes in the network

Note: The "+" after the metric indicates the forward, and the "-" indicates the reverse.

**Table 3:** Metrics system for analyzing the importance of network nodes.

Perspective	Network Metrics	Evaluation
Direct Influence	Out strength (+); In strength (+); Total strength (+)	The strength of the node’s external contact
Intermediate Influence	Weighted Betweenness Centrality (+)	The number of shortest paths through a node
Comprehensive Influence	Kleinberg’s hub centrality scores (+)	The number of authoritative nodes connected by this node

Note: The "+" after the metric indicates the forward, and the "-" indicates the reverse.

#### 3.2.3 Network community detection

To reveal the structural organization of the semiconductor trade network and understand its evolutionary patterns and developmental trends, community detection techniques are employed to partition the network’s nodes into distinct communities. The “cluster Louvain” method, a modularity-based community detection algorithm, is employed for this purpose. This algorithm starts by treating each node as an independent community. It then iteratively reassigns nodes to optimize modularity, merging communities when further gains are no longer possible. This process continues until a single community remains or no further improvements can be achieved. For operational implementation of community detection in the trade network analysis, the "aricode" package in R was utilized.

#### 3.2.4 Network robustness analysis

Network robustness analysis offers a valuable tool for evaluating the stability and resilience of trade networks, thereby illuminating their vulnerabilities and potential disruptions. This paper leverages computer program simulation to directly observe the performance of the global semiconductor trade network under disruptive shocks. To assess the robustness of each segment within the network, two attack methods are employed: random attacks and targeted attacks, focusing on nodes as the targets of disruption. The R programming environment was used to construct random sequence shock models and targeted sequence shock models for robustness analysis.

## 4. Overview of global semiconductor trade

### 4.1. Distinct value chain characteristics

**Fig 1:**
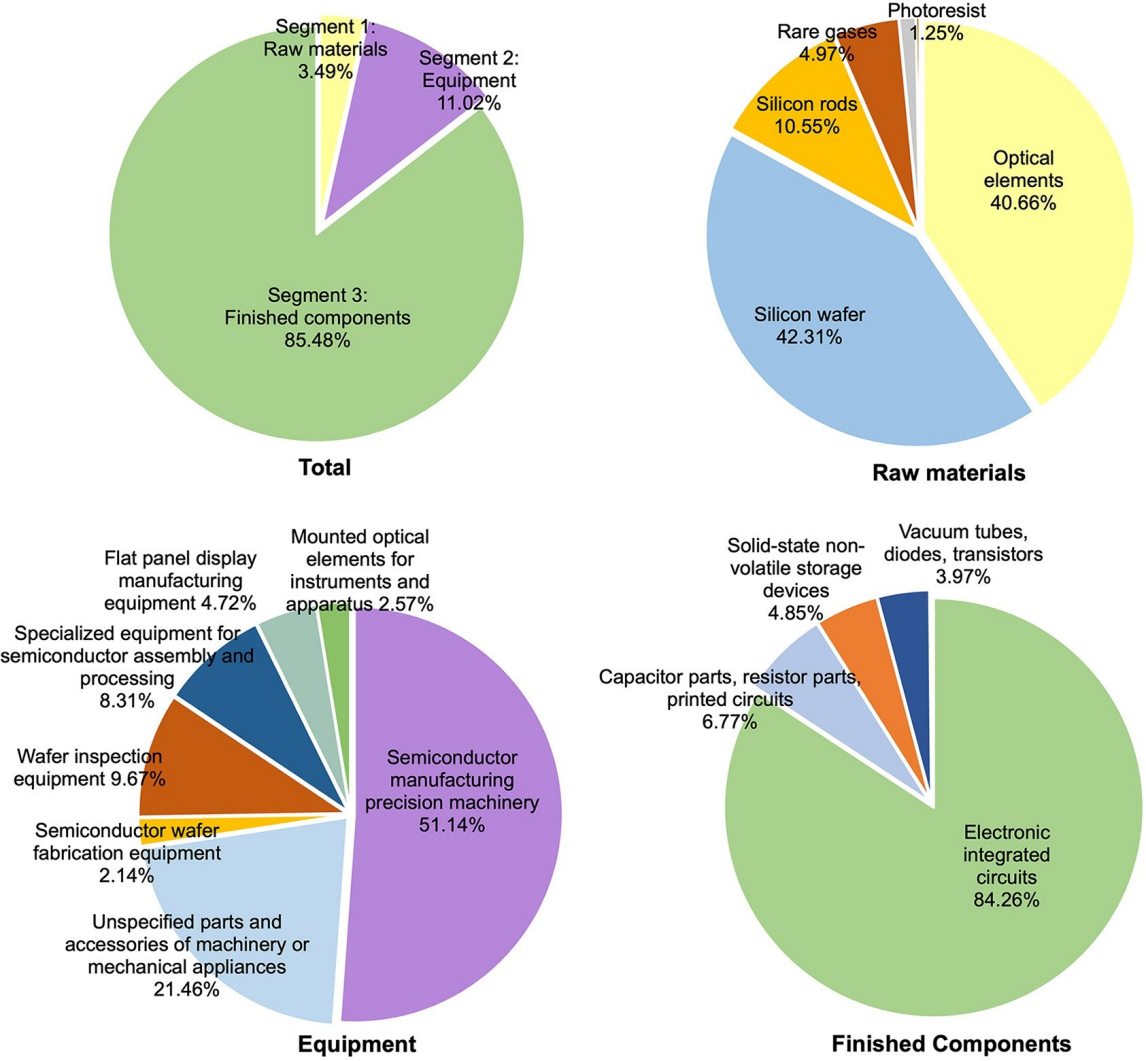
Global trade size of semiconductor industry chain (2021).

An in-depth analysis of the 2021 trade data (Fig 1) reveals significant disparities in trade volumes across the global semiconductor industry chain.

The trade share for semiconductor materials, equipment, and finished components is 3.49%, 11.02%, and 85.48%, respectively. This distribution highlights a clear focus on finished component trade within the industry. Due to the nature of a value chain, some double counting may occur in downstream segment trades when using trade statistics for analysis. This is a normal phenomenon and is credible. The trade of raw materials is concentrated on silicon wafers, optical components, and silicon rods, which collectively account for over 93% of the segment. The composition of equipment trade is relatively dispersed, with key equipment such as Semiconductor manufacturing precision machinery (Photolithography machine, Etching machine, Ion implanter), along with Unspecified parts and accessories of machinery or mechanical appliances, holding respective shares of 51.14% and 21.46%. Wafer inspection equipment and Specialized equipment for semiconductor assembly and processing also exhibit significant shares. The trade of finished semiconductor components is highly concentrated, with Electronic integrated circuits alone accounting for a staggering 84.26%, far exceeding other categories.

### 4.2. Asia, the US, and the EU dominate trade flows

**Fig 2:**
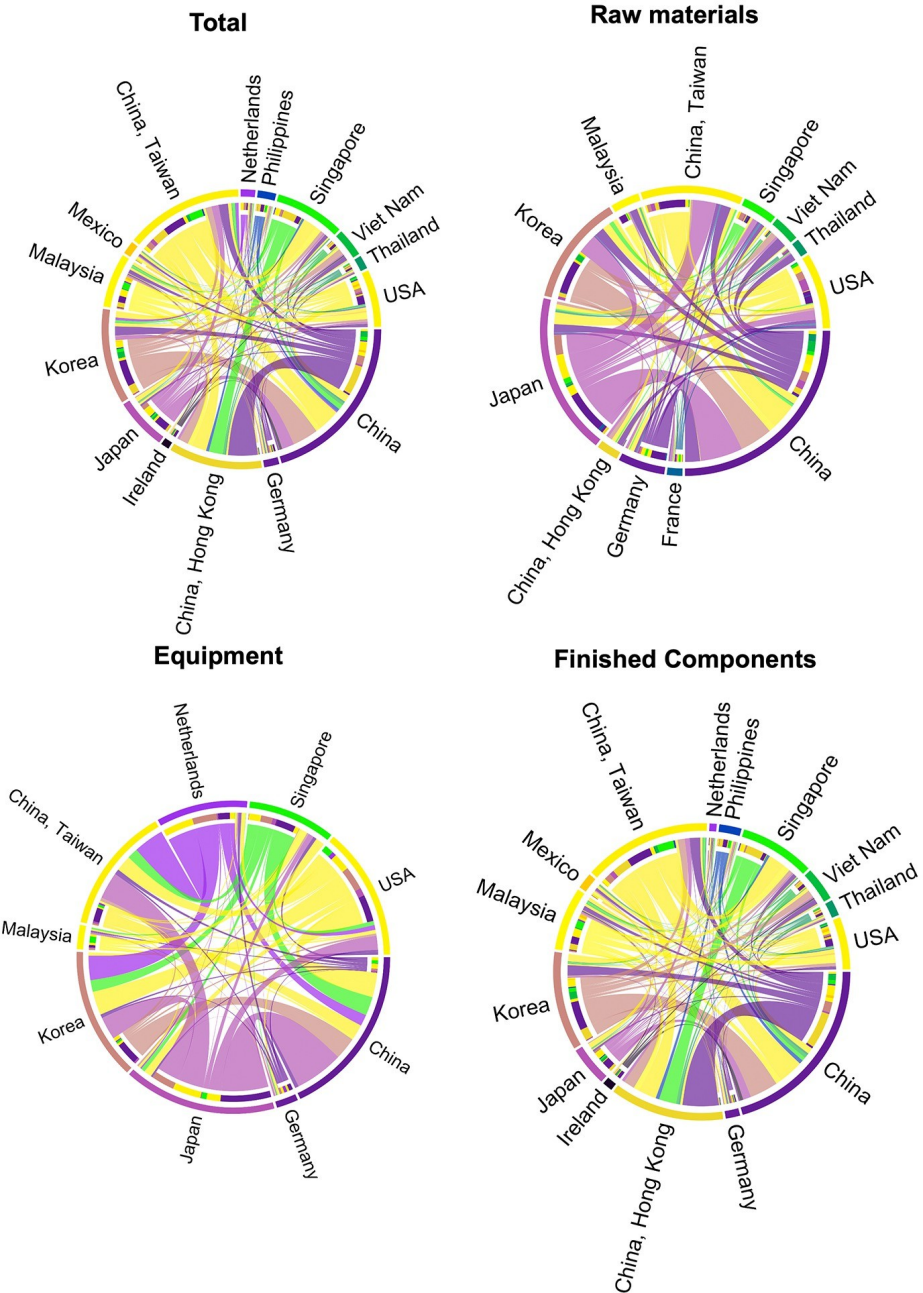
Global semiconductor industry chain trade flows (2021). Note: Trade in thousands of dollars.

The chord diagram visually depicts trade flows between economies, illustrating the global trade network in a circular structure. Each node represents an economy, chords connect them to show their trade relationships. When interpreting the diagram, it is essential to consider the width and color of the chords. The width of a chord reflects the strength of the trade flow, while the direction of the chord’s arrow signifies the direction of the trade. For instance, if there is a chord from country A to country B, it indicates the export from A to B. Consequently, the cumulative width of chords originating from each node signifies the out-strength of that node. Similarly, the total width of chords received indicates the in-strength, and the overall width of all chords associated with a particular node represents the total-strength. By observing the intersections and connections of chords, one can understand the complex trade network and dynamics among countries or regions. The number of nodes in the semiconductor trade network exceeds 200 (Fig 2). To present the trade flow between important nodes more clearly in the chord diagram, this paper selects economies with a relatively high share of total trade for analysis, with a threshold set at the trade share not less than one percent of the total trade within their respective trade networks. Analysis of trade flows in 2021 reveals a concentration of global semiconductor trade in Asia, the US, and select regions within the EU.

The Asian region plays a pivotal role in the semiconductor trade network, with Japan, Chinese Taiwan, Korea, and Singapore emerging as significant global bases for semiconductor production. These regions boast comprehensive and mature semiconductor industry chains encompassing IC design, manufacturing, packaging, and testing. They have a substantial trade share in the three primary sectors of the semiconductor industry, which include semiconductor materials, equipment, and products. Remarkably, Chinese Taiwan and Korea excel in manufacturing and are leaders in exporting manufactured semiconductor products. Conversely, Japan plays a crucial role in exporting materials and equipment, surpassing even the US and the EU. Chinese mainland has made rapid progress in the semiconductor industry, establishing itself as one of the world’s largest semiconductor markets and production bases with a well-rounded industry chain. However, it faces a notable trade deficit as the world’s largest net importer of semiconductor materials and equipment. Additionally, Malaysia, Vietnam, Thailand, and the Philippines have emerged as vital centers for semiconductor packaging and testing, focusing their industry chains and trade activities primarily on manufactured products.

While the US may not be the largest player in total semiconductor trade, it remains a crucial participant in the global semiconductor industry with a well-rounded and balanced industry chain. It exhibits distinct strengths in fundamental research, design, materials, packaging, and testing. In terms of trade, the US holds a significant global share in semiconductor materials and equipment, resulting in a surplus. An analysis of trade flows reveals a strong focus on exporting to the Asian region, including Chinese mainland, Chinese Taiwan, Japan, and Korea, while imports primarily come from Japan and the EU.

In comparison, the EU’s share in global semiconductor trade is relatively modest. Only Germany, the Netherlands, and Ireland contribute more than one percent, without exceeding five percent. Germany and the Netherlands have notable and mature semiconductor industry chains. For instance, Germany is home to globally recognized companies across various segments, including Siemens EDA in semiconductor EDA/IP, Infineon and Bosch in chip design, Linde Gas and BASF in materials, Aixtron in equipment, and X-fab, Core, Siltronic in manufacturing. Germany’s strength lies in manufacturing materials and equipment, making it a crucial net exporter in the semiconductor materials sector. This advantage stems from its dominance in the market for high-purity silicon. Despite silicon’s abundance, only a few suitable deposits, mainly quartz, exist for high-tech applications. Germany possesses ample high-quality quartz sand, strengthening its position in the semiconductor materials trade. Overall, the EU primarily engages in the semiconductor trade’s materials and equipment segment, exhibiting a significant surplus, with Asia being the primary destination for exports.

## 5. Evolution of the semiconductor trade network

### 5.1. Evolution of the overall structure of the trade network

This section explores the size, “small-world” characteristics (a network with short path lengths between most nodes), and connectivity of semiconductor trade networks.

### The size of the semiconductor trade network

In this section, the size of the semiconductor trade network is examined through three metrics: the number of participating countries/regions (nodes), the number of trade relationships between them (edges), and total trade volume (Table 4). In terms of the number of nodes, although the semiconductor materials and equipment trade network has slightly fewer nodes than the finished components trade network, it experienced a modest growth of approximately 9% between 2007 and 2012. Conversely, the number of nodes involved in the trade of finished components showed no significant growth. In terms of the number of edges, the semiconductor materials and equipment trade network has significantly fewer edges than the finished components trade network. The number of edges in the three trade segments experienced a substantial growth of about 35% between 2007 and 2012, followed by a modest growth of about 8% between 2012 and 2017. Between 2017 and 2021, the growth stagnated, with the semiconductor equipment segment even experiencing some decline. The trade volumes of the three segments are compared in Section 3, and the subsequent analysis focuses on examining their temporal changes. The trade volume of the semiconductor materials segment has shown limited growth, with an average annual growth rate of 1.9% from 2007 to 2021. Meanwhile, the semiconductor equipment and finished components segments were in a high-speed growth phase from 2007 to 2021, with average annual growth rates of trade volume reaching 8.17% and 5.48%, respectively. It is worth noting that the fluctuation interval of the trade volume in the three segments is inconsistent. The growth of semiconductor materials mainly occurred in two phases, from 2007 to 2012 and from 2017 to 2021. The growth phase of semiconductor equipment was observed between 2012 and 2021, while the growth phase of finished semiconductor components was evident between 2017 and 2021.

**Table 4:** Metrics of the overall structure of the semiconductor trade network.

Year	Trade network	Num of nodes	Num of edges	Total trade	Diameter	Average path length	Density
2007	segment1	209	3441	328.75	4	2.170	0.079
2012	segment1	217	4730	370.53	4	2.151	0.101
2017	segment1	222	5114	368.55	5	2.159	0.104
2021	segment1	217	5135	427.41	6	2.175	0.110
2007	segment2	207	3532	449.17	4	2.186	0.083
2012	segment2	216	4524	527.96	5	2.113	0.097
2017	segment2	219	4890	906.08	4	2.112	0.102
2021	segment2	215	4783	1347.89	5	2.129	0.104
2007	segment3	223	8135	4951.13	3	1.887	0.164
2012	segment3	226	11077	5119.89	4	1.821	0.218
2017	segment3	226	12025	6959.83	3	1.791	0.236
2021	segment3	226	12089	10455.12	4	1.797	0.238

Note: “Total trade” in billions of dollars, the description of other indicators can be found in Table 2.

### The “small-world” characteristics of the semiconductor trade network

In this section, the “small-world” characteristics of trade networks are examined by analyzing two metrics: network diameter and minimum path length [32]. The network diameter signifies the longest distance between any two nodes within the network. The analysis presented in this paper demonstrates that the global semiconductor trade network displays distinct "small-world" characteristics within its different segments, encompassing semiconductor materials, equipment, and finished components. The intensity of these characteristics varies, with a weaker presence observed in semiconductor materials, followed by equipment, and the strongest in finished components. In 2021, the semiconductor materials trade network displayed a decreasing trend in "small-world" features, with the maximum distance between any two nodes reaching six edges, thereby requiring traversing five nodes to establish a connection. Conversely, during the same period, the maximum distances in the semiconductor equipment and finished components networks were five and four edges, respectively. Furthermore, the minimum path length, representing the average shortest distance between nodes, serves as a metric of network accessibility. The minimum path lengths of semiconductor materials, equipment, and finished components in the global semiconductor trade network were approximately 2.2, 2.1, and 1.8, respectively. These values align with the findings of the analysis based on the farthest distance and further validate the unique "small-world" characteristics of the semiconductor trade network.

### The connectivity of the semiconductor trade network

In this section, the connectivity of the trade network is assessed by measuring network density, which quantifies the ratio between the actual number of edges and the maximum potential number of edges within the network. In large-scale networks, the number of possible edges increases exponentially as the number of nodes grows, resulting in a decrease in network density and a sparse network. However, the global semiconductor trade network demonstrates a notably high level of connectivity across all segments, particularly in the semiconductor material and equipment trade network, which exhibits similar node numbers and closely aligned network density values. Notably, the network density rises with the increase in node count, indicating robust connectivity between nodes in the semiconductor materials and equipment trade network, diverging from the traditional sparsity characteristic. In contrast, the connectivity of the finished semiconductor components trade network presents a more pronounced pattern. From 2007 to 2021, the density of the finished semiconductor components trade network increased from 0.164 to 0.238, highlighting its strong connectivity compared to the general sparsity observed in large-scale networks.

### 5.2. Evolution of node influence

This section analyzes the impact of individual nodes within the semiconductor trade network using a multifaceted approach. Our primary objective is to elucidate the roles played by individual economies within the network and offer micro insights into the intricate architecture of the trade network.

### The direct influence of nodes in the semiconductor trade network

In this section, the direct influence of nodes in the network is quantified by assessing out-strength and in-strength, weighted by trade volume. The top 20 nodes were identified based on their total-strength, illustrating their pivotal role in controlling interactions between other nodes. Key findings include: (1) Emerging economies have notably increased their influence within the semiconductor trade network (S1 Fig). In 2007, Chinese mainland led in import trade volume but exhibited comparable export trade volume to Singapore, lagging behind Chinese Taiwan, Japan, and the US. In 2012, Chinese mainland had surpassed Japan in export volume, ranking second only to Chinese Taiwan. Then, in 2021, Chinese mainland exceeded Chinese Taiwan in semiconductor export volume and emerged as a global leader. During the same period, Korea’s semiconductor exports grew rapidly, moving from sixth in the world in 2007 to the third in 2021. Special attention is warranted for Southeast Asian economies, particularly Malaysia, which has achieved significant growth in semiconductor exports and established itself as a key player in the economy’s manufacturing sector. (2) While traditional powerhouses such as Japan, the United States, and Germany maintain a leading influence in the semiconductor materials trade network, the growth of emerging powers can no longer be overlooked (S2 Fig). Historically, Japan, the US, and Germany have long been leaders in semiconductor raw materials export trade, especially Japan, which covers a comprehensive range of semiconductor materials such as silicon wafers, mask plates, electronic special gases, CMP materials, and photoresists. However, with the remarkable progress of the semiconductor industries in regions such as mainland China, South Korea, and Chinese Taiwan, the export volume of semiconductor materials from these regions is gradually catching up and even surpassing that of the US. This signifies the ongoing evolution of global semiconductor industry dynamics, underscoring the competitive challenges faced by traditional industry leaders. (3) The absolute dominance of Japan, the US, and the Netherlands in the semiconductor equipment trade network is evident (S3 Fig). These countries have consistently dominated semiconductor equipment exports. Japan, in particular, leads in thin film deposition, etching, photolithography, and other manufacturing equipment, holding approximately 30% of the market share in 2022. The Netherlands, as a crucial hub for the European semiconductor industry, boasts a complete value chain and a robust ecosystem in equipment manufacturing. Notably, ASML, the world’s exclusive supplier of extreme ultraviolet lithography (EUV), monopolizes the high-end lithography market, which is a critical component in the semiconductor industry chain. Additionally, the Netherlands is home to renowned packaging equipment manufacturer Besi and photonic chip manufacturer Smart Photonics. (4) The influence of the Asian region in the trade network of finished semiconductor components has long surpassed that of Europe and the US (S4 Fig). The export trade volume of finished semiconductor components reflects a region’s capacity for processing and manufacturing semiconductor components. Several economies in the East and Southeast Asian region have significantly increased their influence in the overall network through advancements in this sector. In 2021, among the top 10 economies in terms of export trade value of finished semiconductor components, 8 were from Asia.

### The indirect influence of nodes in the semiconductor trade network

In this section, the indirect influence of nodes in the network is quantified by assessing the betweenness centrality, weighted by trade volume. The top 20 nodes were identified based on their total-strength, illustrating their pivotal role in controlling interactions between other nodes. In a network, a node occupying a position on the path between multiple other nodes is deemed crucial as it can control interactions among them. The overall patterns of the semiconductor trade network, material trade network, equipment trade network, and finished components trade network have remained relatively robust (S5–S8 Figs). However, a notable increase in betweenness centrality was observed for emerging economies, particularly Chinese mainland. Taking the semiconductor material trade network as an example, Chinese mainland’s betweenness centrality ranked fourth globally in 2007, with a substantial gap compared to the US, Japan, and Germany. Nevertheless, by 2012, it had surpassed Japan to claim the second position worldwide, and by 2017, it had even surpassed the US to secure the first rank globally.

### The comprehensive influence of nodes in the semiconductor trade network

In this section, the comprehensive influence of nodes in the network is quantified by assessing the hub centrality (Kleinberg’s hub centrality scores, grounded in the Hyperlink-Induced Topic Search algorithm), weighted by trade volume. The top 20 nodes were identified based on their total-strength, illustrating their pivotal role in controlling interactions between other node. Key findings include: (1) The Asian region holds unparalleled importance in the overall trade network, as depicted in S5 Fig. In both the 2007 and 2012 rankings, eight out of the top 10 economies were from Asia. Furthermore, in the 2017 and 2021 rankings, Thailand has surpassed Germany, with the US being the only non-Asian country within the top 10 economies. While Chinese Taiwan and Japan have maintained their leading positions for an extended period, in recent years, economies such as Korea, Chinese mainland, and Singapore have gradually surpassed Japan in influence. (2) In the materials trade network, the combined influence of Japan, Korea, and Chinese Taiwan has consistently remained within the top three positions globally since 2012, forming the first tier (S6 Fig). On the other hand, Germany, the US, and Chinese mainland constitute the second tier. (3) In the equipment trade network, Japan, the US, and the Netherlands have maintained their positions in the first echelon over an extended period (S7 Fig). Furthermore, there has been a rising influence of Asian economies such as Singapore, Korea, and Chinese Taiwan, albeit with a considerable gap still present. (4) The Asian region occupies a central role in the trade network of finished components (S8 Fig). Chinese Taiwan has consistently held a nearly unrivaled comprehensive influence globally. However, with the development of semiconductor manufacturing in Korea and Chinese mainland, by 2021, Chinese Taiwan, Chinese mainland, and Korea constitute the first echelon of the global semiconductor parts and components trade network, leaving Singapore, Malaysia, Japan, and others significantly behind.

### 5.3. Community structure and evolution in networks

The community structure of trade network is crucial for unraveling the complex dynamics of global trade. This analysis reveals the clustering of economic activities, illuminating the formation, evolution, and dynamics of these clusters and enhancing our understanding of trade patterns. To clearly depict the community structure within the trade network, trade flows valued below $5 million were excluded. The community structure is identified through multi-level optimization of modularity [33] (Table 5). Specifically, the default resolution (1.0) is employed for the community detection. Notably, from igraph version 1.3 onwards, this method introduces a random element by processing vertices in random order during each iteration. This randomization can potentially influence the consistency of the detected communities across different executions of the algorithm. The community detection process was repeated 50 times to address this potential variability. The consistently high Normalized Mutual Information (NMI) values exceeding 0.88 indicate the stability of the community detection. Moreover, experiments were conducted by varying the resolution parameter and repeating the community detection 50 times for each resolution value. The average NMI values for resolutions of 0.9, 0.8, 0.7, 0.6, and 0.5 were 0.92, 0.91, 0.85, 0.78, and 0.63, respectively, further demonstrating the stability of the community detection results.

**Table 5:** Community structure of the semiconductor trade network.

Year	Raw materials			Equipment			Finished components		
	Trade share of the community (%)		Modularity Value	Trade share of the community (%)		Modularity Value	Trade share of the community (%)		Modularity Value
2007	48.09 31.52 15.98	2.87 1.53	0.56	36.08 31.08 22.41	5.35 5.03	0.55	24.01 20.76 19.99	18.32 14.36 2.28	0.55
2012	27.59 26.49 14.32 13.81	9.57 6.66 1.42	0.60	22.11 19.53 19.46	14.77 12.10 11.80	0.58	29.63 25.07 11.86 11.52	9.44 7.30 3.07 2.10	0.57
2017	67.97 18.79 4.90	3.98 3.85	0.52	32.42 27.66 17.94 9.50	9.15 2.10 1.14	0.66	44.39 21.94 12.65 7.14	5.73 4.00 3.33	0.57
2021	29.15 26.46 21.89	19.82 2.68	0.45	46.83 30.90 16.23	3.75 1.91	0.57	52.80 18.68 14.14	6.81 5.27 2.03	0.63

Note: “Trade share of the community” refers to the trade share of each community, and communities with trade volume shares below 1% have been removed from the analysis.

To assess the relative importance of different communities within the trade network and observe the distribution of power among these communities, the semiconductor trade volume of all members within each community was aggregated (including trade with members outside the community). As evident in Table 5, the modularity values for the detected communities all exceed 0.45. This indicates that the networks have well-defined community structures, nodes within the same community have denser connections among themselves compared to nodes in different communities.

The overall structure of the raw materials trade network transitions from centralization to diversification. By the end of the observation period, the network comprises four communities of comparable size, with the trade volume share of the largest community not exceeding 30%. In contrast, both the equipment trade network and the finished components trade network exhibit a “polarization” trend, characterized by the “strong getting stronger and the weak getting weaker.” Specifically, the trade volume share of the largest community in the equipment trade network increased from 36.08% in 2007 to 46.83% in 2021. The polarization trend is even more pronounced in the finished components trade network, where the trade volume share of the largest community rose from a mere 24.01% in 2007 to 52.8% in 2021. In summary, the raw materials trade network exhibits a diversification trend in recent years, while the equipment and finished components trade networks remain in a state of persistent “polarization.”

## 6. The robustness of network

**Fig 3:**
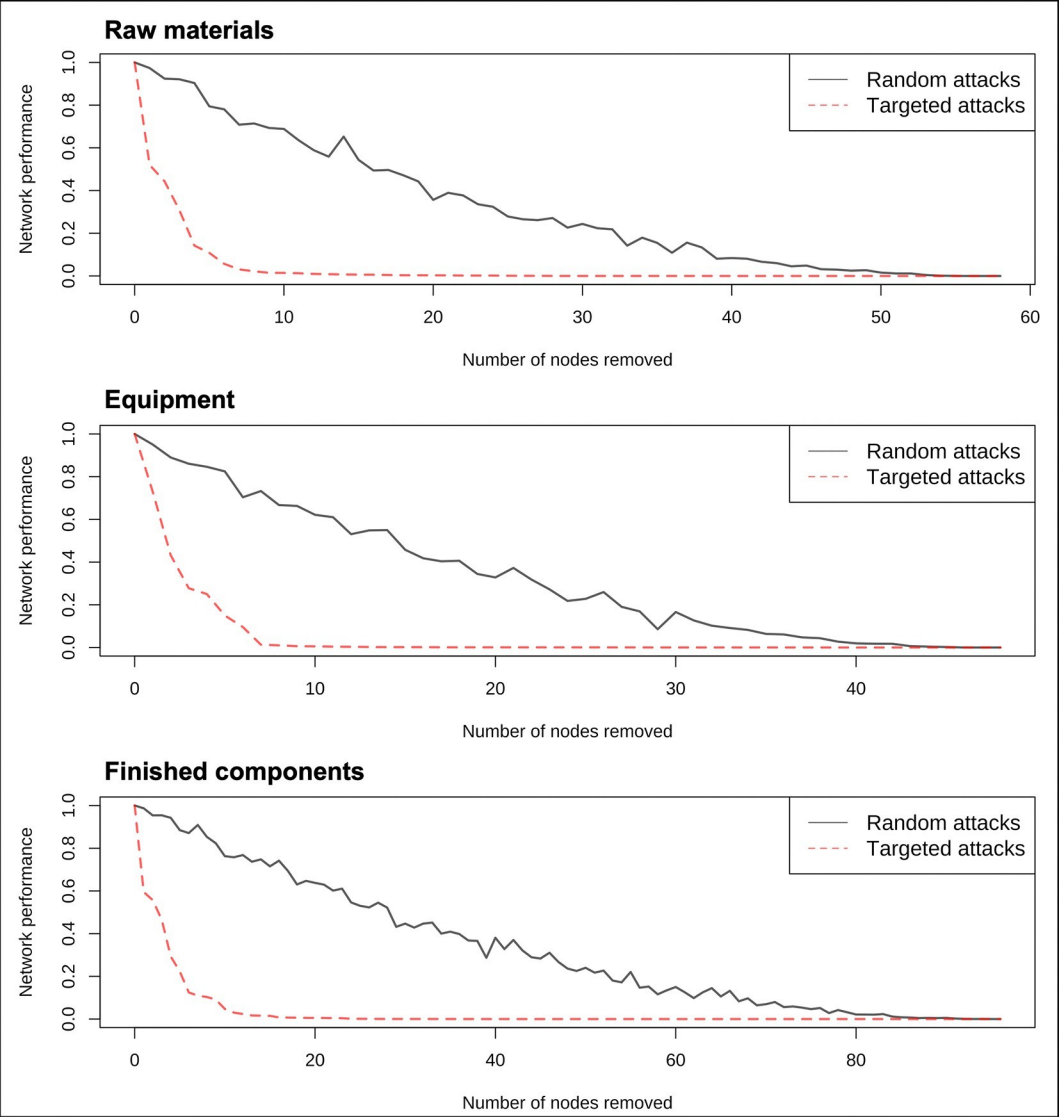
Robustness of semiconductor trade networks under random and targeted attacks (2021).

Referring to Lou et al. [34], this paper employs posterior measures to study the robustness of the semiconductor trade networks. Specifically, this paper utilizes computer program simulations to evaluate the ability of the semiconductor trade networks to maintain essential functions under disruptions. Network performance is measured by comparing the trade volume of the post-attack network to that of the original network. Two types of attacks are considered: random attacks and targeted attacks. Random attacks are conducted by removing nodes using the Mersenne-Twister algorithm, whereas targeted attacks remove nodes in descending order of their strength centrality. To mitigate randomness on the shock simulation results, 50 simulations were conducted for each network and averages were calculated. Consequently, the line representing network efficiency under random attacks in Fig 3 exhibits fluctuations. In this paper, a network performance metric value below 0.1 is defined as a “collapse state”, indicating that only 10% of the original trade volume remains in the network. The term "breaking point" refers to the number of nodes that must be removed to induce a "collapse state."

As depicted in Fig 3, the slope of the line representing targeted attacks is considerably steeper than that of random attacks. This observation implies that semiconductor trade networks exhibit greater stability under random attacks compared to targeted attacks. Corroborating this finding, Table 6 reveals that the number of nodes required to reach the breaking point under random attacks is approximately six times higher than that under targeted attacks for semiconductor trade networks. For instance, the raw materials trade network in 2021 comprises 58 nodes. Under random attacks, removing 39 nodes would cause the network to collapse, while under targeted attacks, only 6 nodes would need to be removed to induce collapse. Examining network size and breaking point, it is evident that the robustness of semiconductor trade networks against both random and targeted attacks increases with the number of nodes in the network. Among the different segments, the finished components trade network exhibits the largest size and the highest robustness against both random and targeted attacks. In contrast, the raw materials and equipment trade networks have smaller sizes and correspondingly lower robustness against both types of attacks.

**Table 6:** Breaking point of semiconductor trade networks under random and targeted attacks.

Year	Raw materials			Equipment			Finished components		
	Number of nodes	Breaking point		Number of nodes	Breaking point		Number of nodes	Breaking point	
		Random attack	Targeted attack		Random attack	Targeted attack		Random attack	Targeted attack
2007	43	29	4	43	30	6	88	59	9
2012	49	32	5	46	31	6	98	65	9
2017	53	36	5	43	31	6	106	74	11
2021	58	39	6	48	33	6	96	67	9

To ensure the robustness of the shock simulation results, this paper conducted a comprehensive set of robustness checks. These checks involved employing alternative attack strategies and additional network performance metrics to assess the sensitivity of the findings. (1) Alternative attack strategies: Random attacks were conducted using two well-established algorithms (the Wichmann-Hill algorithm and the L’Ecuyer-CMRG algorithm). Additionally, targeted attacks were based on node degree centrality, a widely recognized measure of node importance within a network. (2) Additional network performance metrics: Beyond the trade volume in the network, this paper also considered average path length and density as supplementary measures. These additional metrics provide insights into the overall connectivity and efficiency of the network under different attack scenarios.

The results obtained from these robustness checks were consistent with the conclusions presented in the previous sections. This consistency across different attack strategies and performance metrics reinforces the validity of the findings and demonstrates that they are not sensitive to the specific choices made in the methodology.

## 7. Discussion

This paper aims to investigate the characteristics and robustness of the global semiconductor trade network from an industrial chain perspective:

1. Dominant global semiconductor trade flows are concentrated in a few key regions: Asia, the US, and the EU. Asia stands out as a central player, with Japan leading in the trade of semiconductor materials and equipment. Chinese mainland, Chinese Taiwan, and Korea possess distinct strengths in the trade of finished semiconductor components. Meanwhile, the US and the EU, retain a significant presence in the trade of semiconductor equipment.
2. The semiconductor trade network continues to grow, with the finished components segment leading at an impressive annual average growth rate of 8.17%. The equipment trade network follows closely at 5.48%, while the raw materials trade network exhibits the slowest growth at 1.9%. Interestingly, all segments exhibit pronounced "small-world" characteristics, deviating from the typical "sparsity" observed in most large networks. This paper’s network density is demonstrably higher compared to Zhang and Zhu [18], likely due to the inclusion of nearly 30 key commodities, enhancing comprehensiveness and accuracy.
3. When evaluating the direct influence of nodes in the network using weighted out-strength and weighted in-strength, Japan, the US, and the EU have consistently held top positions in the raw materials and equipment trade networks. In the finished components trade network, Chinese Taiwan and Korea have long been at the forefront, while Chinese mainland has caught up, and Southeast Asian economies, represented by Malaysia, are accelerating their pursuit. Measuring the indirect influence of nodes in the network using weighted betweenness centrality, the semiconductor trade network structure shows stability, with the main change being Chinese mainland’s rising influence (betweenness centrality). Evaluating overall influence (Hub centrality), Japan, Korea, and Chinese Taiwan lead in raw materials, Japan, the US, and Netherlands in equipment, and Chinese Taiwan, Korea dominate finished components, with Chinese mainland catching up. These findings align with Ren et al. [17]: traditional powers (Japan, the US, Korea, Germany) hold the lead in raw materials, but Chinese mainland is making strides, influencing Southeast Asia (Malaysia, Vietnam, Thailand). Equipment remains dominated by traditional players, while East and Southeast Asia are the new forces in finished components.
4. Compared to Ren et al. [17] and Zhang and Zhu [18], this paper adds a community structure analysis section, which finds that the raw materials trade network in the global semiconductor industry chain has shown a diversification trend in recent years, while the equipment and finished components trade networks are in a state of persistent “polarization.”
5. The semiconductor trade network exhibits a typical characteristic: remarkably robust against random attacks yet highly vulnerable to targeted attacks. As the network scales, the robustness of each segment generally strengthens. Among segments, the finished components network, boasting the largest size, demonstrates superior robustness against both random and targeted attacks. Compared to networks like petroleum [9] and pesticides [12], the semiconductor network exhibits greater robustness under random attacks, but suffers more under targeted attacks. Compared to Zhang and Zhu [18], the semiconductor trade network constructed in this paper shows greater robustness under random attacks, possibly due to the limited number of commodity categories selected in that paper. Under targeted attacks, both studies found that the upstream segments exhibit weaker robustness under targeted attacks.

Centralization in the global semiconductor supply chain boosts efficiency and innovation through economies of scale and technological specialization. However, this concentration also introduces systemic risks, such as vulnerability to geopolitical tensions, natural disasters, and trade policy shifts. The 2021 chip shortage, which disrupted automakers like General Motors, Ford, and Volkswagen, highlights these risks. Moreover, market concentration can stifle competition and innovation. To mitigate these long-term drawbacks and ensure supply chain resilience and industry competitiveness, companies and policymakers must prioritize strategic diversification. This encompasses geographic diversification, supplier diversification, investment in alternative technologies, and the adoption of flexible supply chain management strategies. Additionally, government support and international cooperation are critical to reduce dependence on single markets or technologies, enhance responsiveness to market fluctuations and external shocks, and ultimately, guarantee the stability and sustainable development of the global supply chain.

The semiconductor trade exhibits a stark polarization. Notably, equipment, dominated by established players like the US, Japan, and the EU, presents a significant bottleneck for new entrants. Stringent export controls and high technical hurdles erected by these nations severely restrict technology access for emerging economies, impeding their technological advancement and innovation. This enforced dependence on foreign technology compels emerging economies to ramp up domestic R&D and innovation investments. While a short-term burden, this pressure can become a crucial catalyst for fostering indigenous technological autonomy and innovation capabilities, ultimately reducing reliance on advanced foreign technologies. Meanwhile, in the finished components segment, Chinese mainland, Korea, and Chinese Taiwan have begun to rise in the global market. However, their ascent is hampered by technical and market barriers imposed by incumbents. These barriers include exorbitant technology licensing fees, intricate international standards, and stringent certification requirements, all acting as formidable entry barriers. Undeterred, these emerging economies are countering these challenges by bolstering domestic R&D investments and forging international collaborations. They are strategically carving out market niches in application-specific integrated circuits (ASICs) and specific application domains to enhance their technological prowess and market responsiveness. Through these concerted efforts, emerging economies aspire to establish robust independent R&D capabilities, ultimately reducing their dependence on developed economies and securing a more advantageous competitive position within the global semiconductor landscape.

In the future, the meteoric rise of quantum computing and next-generation semiconductor technologies threatens to exacerbate the existing polarization within the semiconductor industry, particularly in terms of technological control and market dominance. Tech-forerunners like the US and China leverage their mastery of core technologies and influence over standard-setting to maintain their leadership in the global market. This technological edge not only grants them a competitive advantage but also empowers them to influence market access and technological development in other regions through strategic licensing and export controls. Furthermore, the burgeoning complexity of these emerging technologies acts as a formidable entry barrier, potentially stifling new competition and fostering further consolidation within the industry. Additionally, the geopolitical spotlight on core technology supply chains has prompted governments to implement stringent export controls on sensitive technologies. This could lead to a reconfiguration and potential regionalization of global supply chains. However, some emerging economies see an opportunity to leapfrog traditional technology segments by directly adopting disruptive technologies like quantum communication. This strategy holds the potential for not only rapid market expansion in specific technology domains but also for carving a niche within the global technological landscape. As a consequence, the future semiconductor trade network will likely undergo a transformation, with trade flows potentially gravitating towards technologically advanced hubs. In the equipment segment, established players like the US and Japan may solidify their dominance, while China could emerge as a new trade center. Moreover, new technologies could trigger a geographical redistribution of production bases and critical R&D centers, potentially shifting these activities towards regions with robust technology policy support. This evolving landscape necessitates that governments and enterprises not only rapidly adapt to technological disruptions but also actively engage in global cooperation and competition. By doing so, they can optimize their strategic positioning and resource allocation within the future economic landscape.

## Supporting information

S1 Fig Nodes’ out-strength and in-strength—Overall network. Note: “Strength” in thousands of dollars.
(TIF)

S2 Fig Nodes’ out-strength and in-strength—Segment 1 raw materials. Note: “Strength” in thousands of dollars.
(TIF)

S3 Fig Nodes’ out-strength and in-strength—Segment 2 equipment. Note: “Strength” in thousands of dollars.
(TIF)

S4 Fig Nodes’ out-strength and in-strength—Segment 3 finished components. Note: “Strength” in thousands of dollars.
(TIF)

S5 Fig Betweenness centrality and hub centrality of nodes—Overall network. (TIF)

S6 Fig Betweenness centrality and hub centrality of nodes—Segment 1 raw materials. (TIF)

S7 Fig Betweenness centrality and hub centrality of nodes—Segment 2 equipment. (TIF)

S8 Fig Betweenness centrality and hub centrality of nodes—Segment 3 finished components. (TIF)
