# Supplementary figures and images for: Analysis of the structure and robustness of the global semiconductor trade network
Source: PLoS One. 2025 Jan 9;20(1):e0313162. doi: 10.1371/journal.pone.0313162 (PMC11717270; doi:10.1371/journal.pone.0313162)

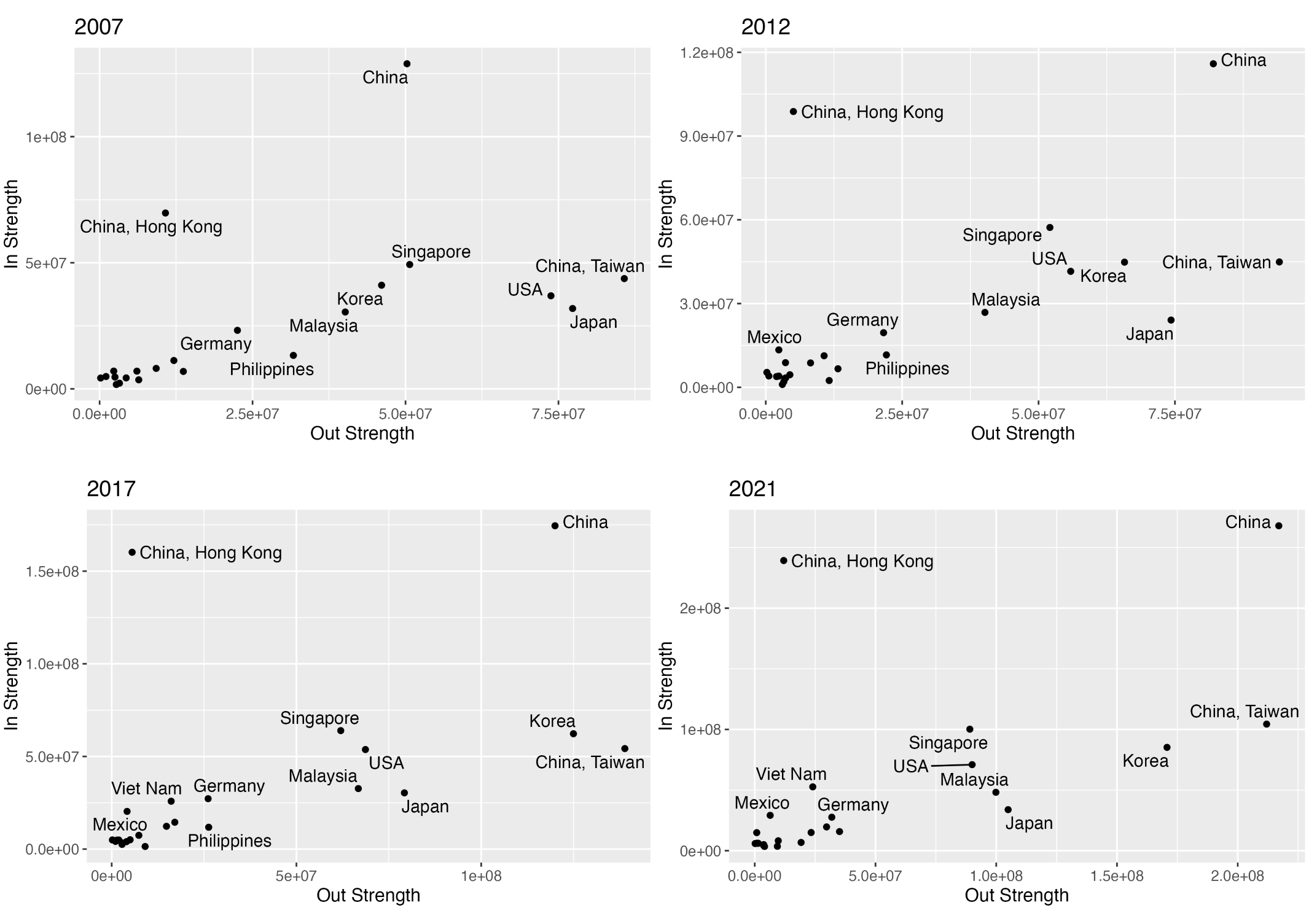

Supplement: S1 Fig — Note: “Strength” in thousands of dollars. (TIF) [file pone.0313162.s001.tif]

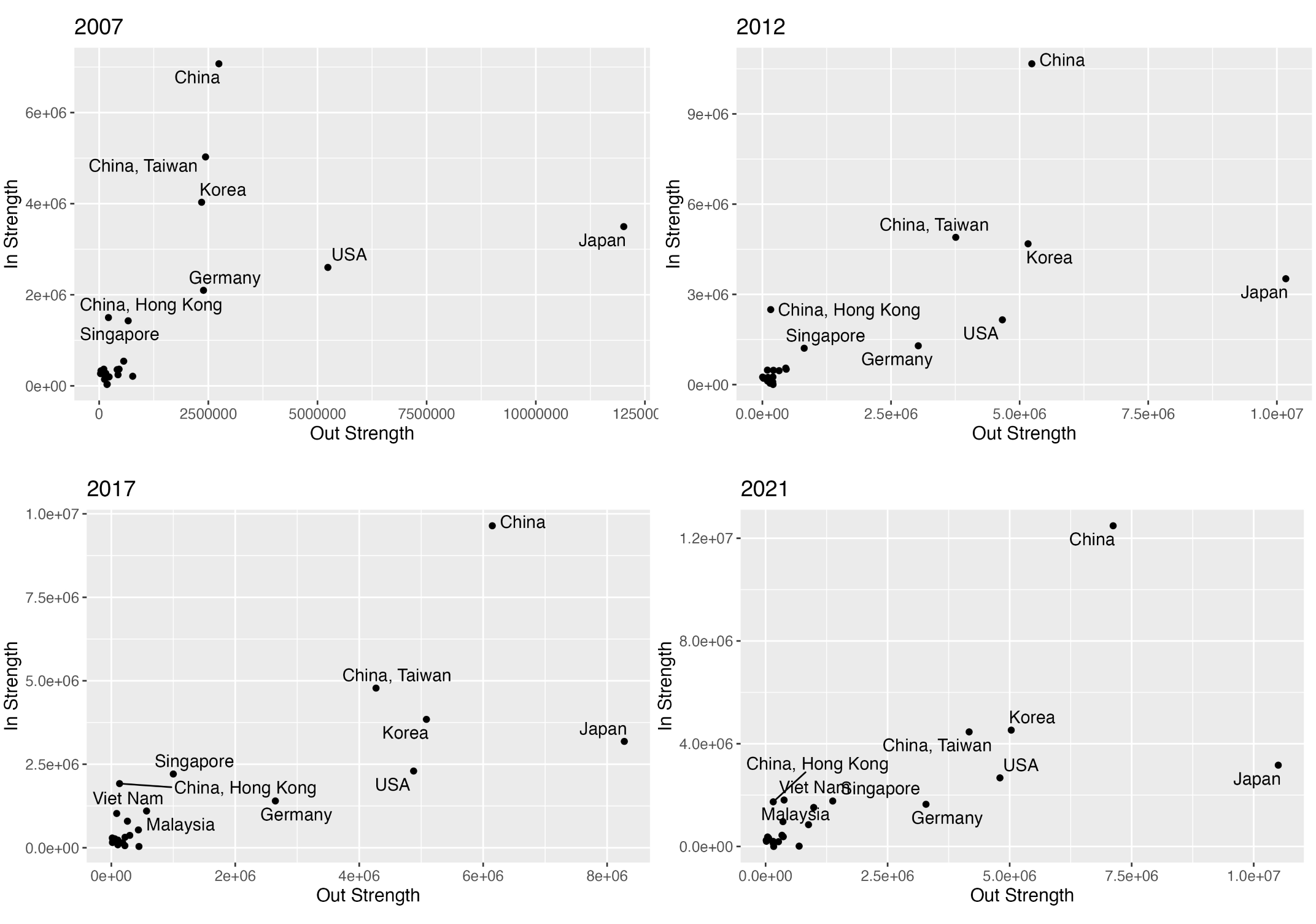

Supplement: S2 Fig — Note: “Strength” in thousands of dollars. (TIF) [file pone.0313162.s002.tif]

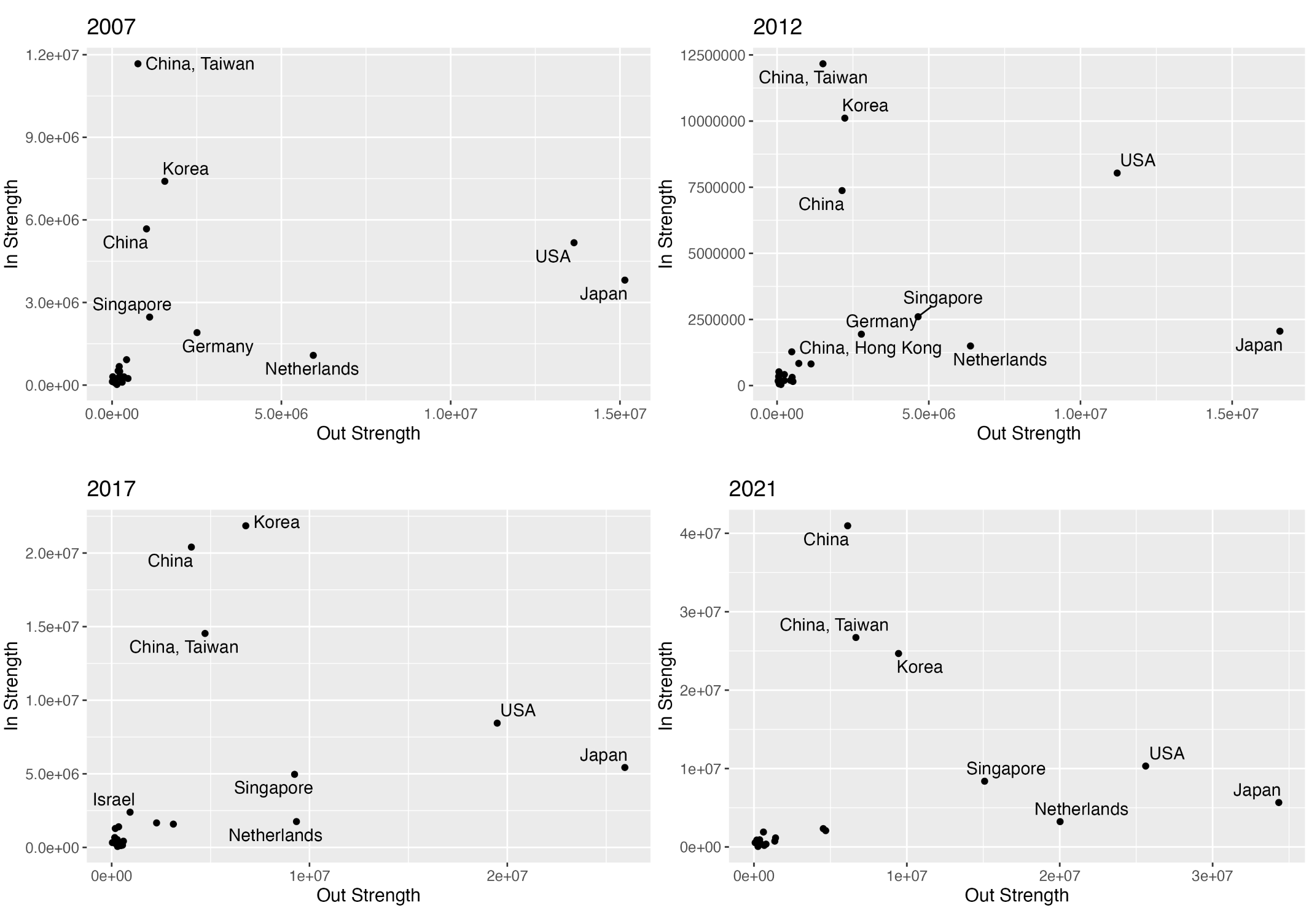

Supplement: S3 Fig — Note: “Strength” in thousands of dollars. (TIF) [file pone.0313162.s003.tif]

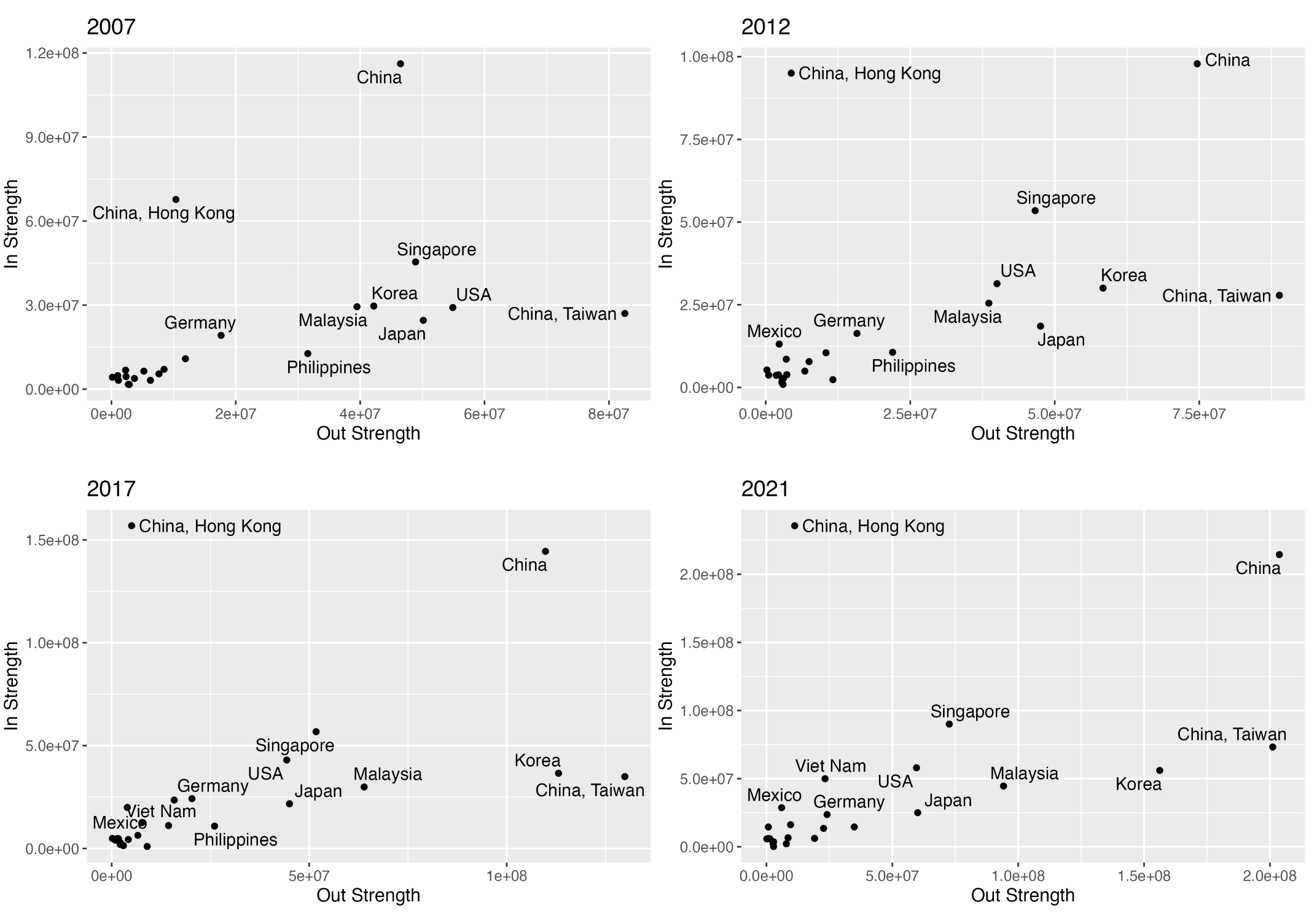

Supplement: S4 Fig — Note: “Strength” in thousands of dollars. (TIF) [file pone.0313162.s004.tif]

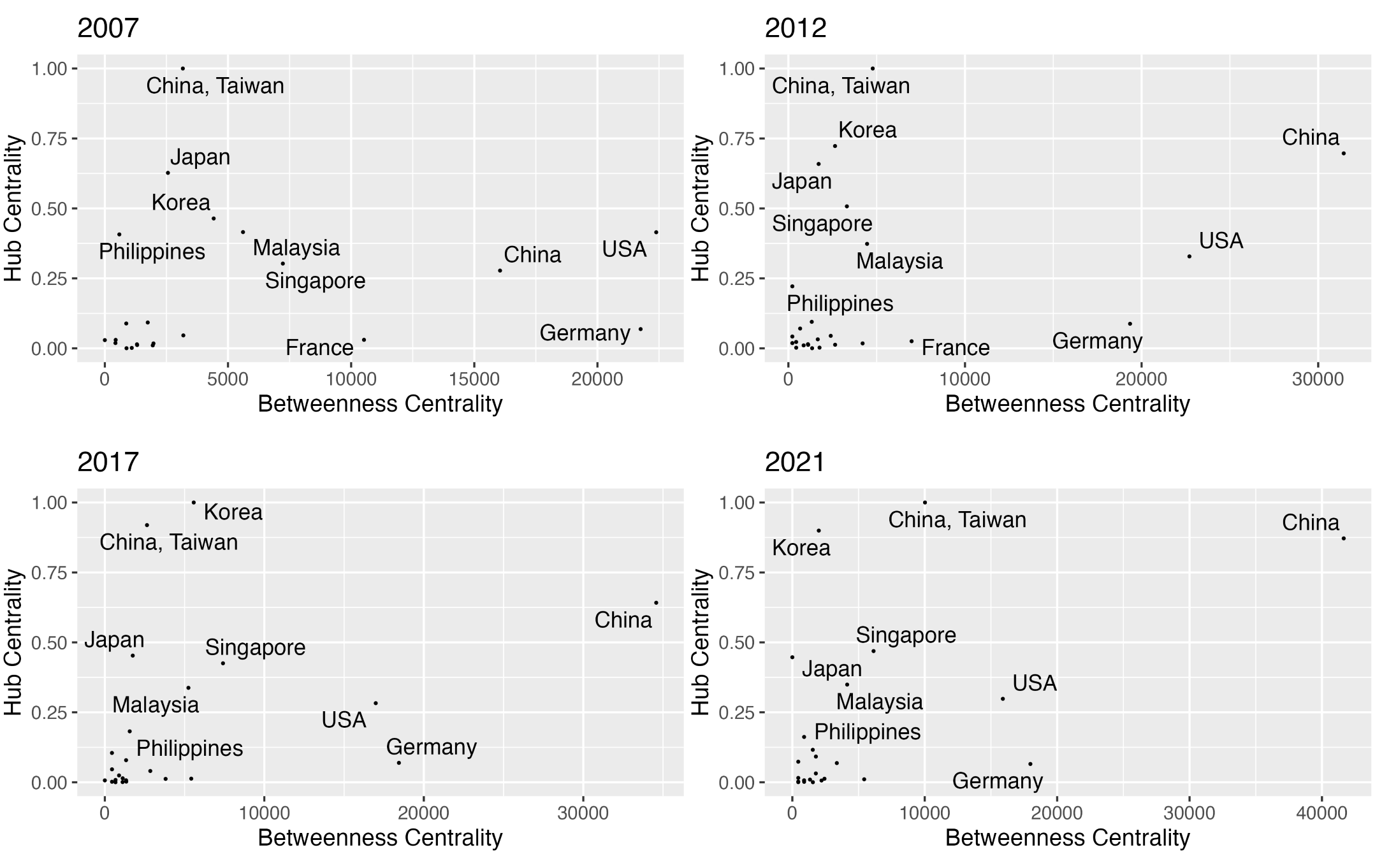

Supplement: S5 Fig — (TIF) [file pone.0313162.s005.tif]

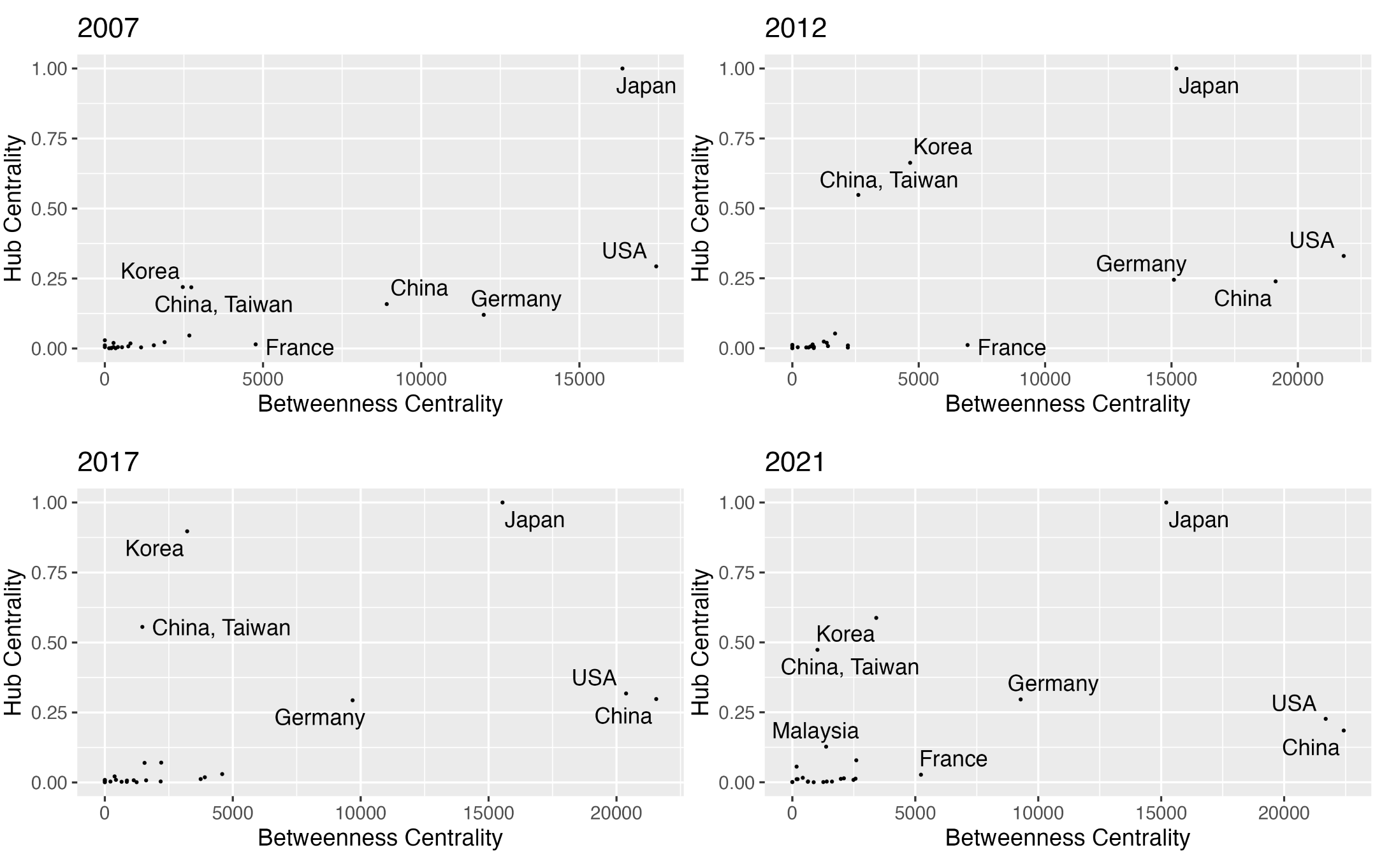

Supplement: S6 Fig — (TIF) [file pone.0313162.s006.tif]

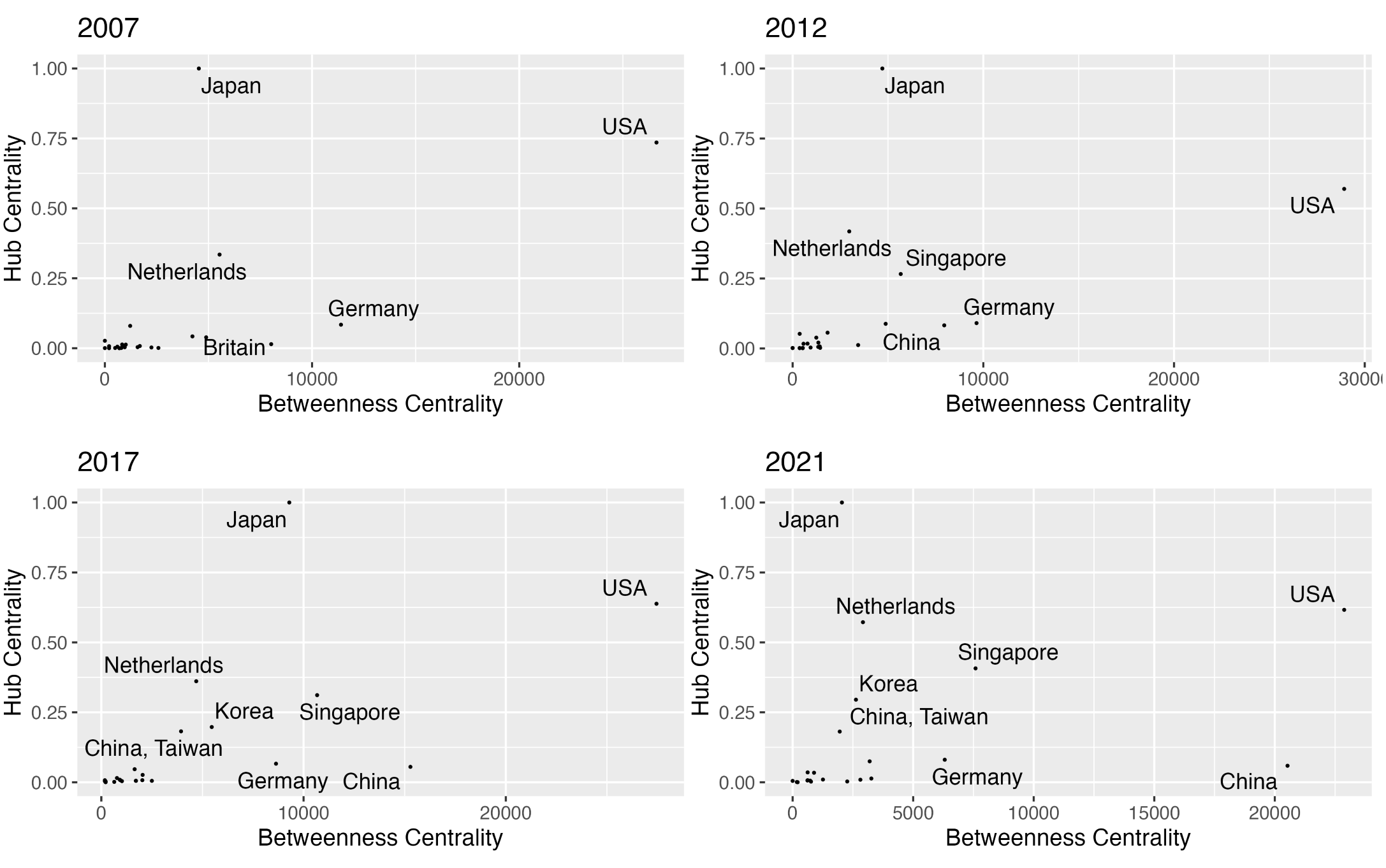

Supplement: S7 Fig — (TIF) [file pone.0313162.s007.tif]

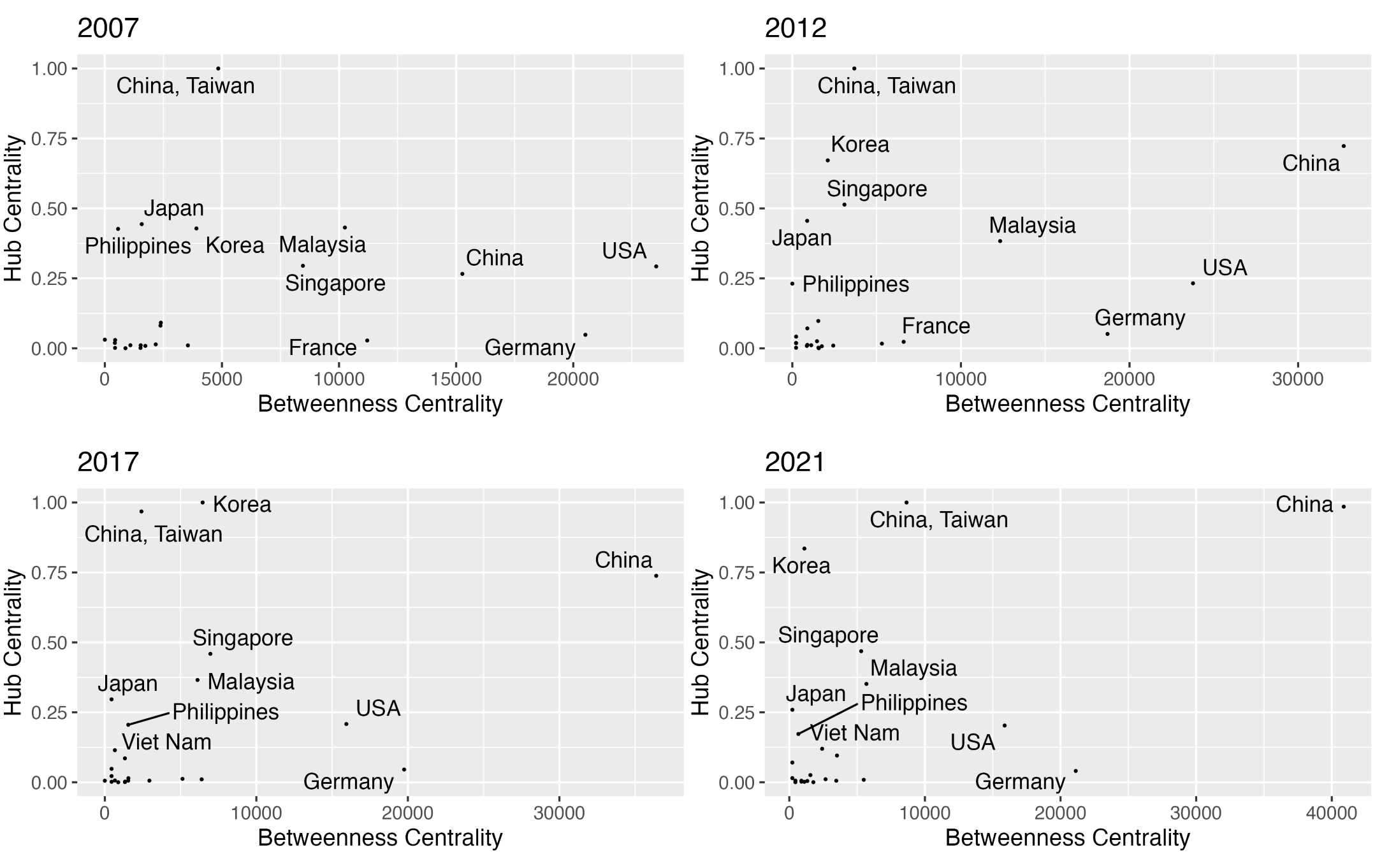

Supplement: S8 Fig — (TIF) [file pone.0313162.s008.tif]
